# Spontaneous resolution of vitreomacular traction following deep scuba diving: a case report

**DOI:** 10.1186/s13256-026-06100-0

**Published:** 2026-06-03

**Authors:** Iden Amiri, Aya Barzelay-Wollman

**Affiliations:** 1 Anschutz School of Medicine, University of Colorado, Aurora, USA; 2 Jules Stein Eye Institute, University of California, Los Angeles, USA

**Keywords:** Vitreomacular traction, Posterior vitreous detachment, Scuba diving, Barometric pressure, Intraocular pressure

## Abstract

**Background:**

Vitreomacular traction is characterized by incomplete posterior vitreous detachment, wherein persistent adherence of the vitreous to the macula results in tractional distortion and associated visual symptoms. Although spontaneous resolution of vitreomacular traction may occur, surgical intervention is typically pursued when significant visual impairment occurs. The mechanism by which spontaneous release of vitreomacular traction is unclear, as there are numerous intrinsic and extrinsic factors that affect the eye. This case highlights the potential effects of sudden changes in barometric pressure on vitreoretinal dynamics.

**Case presentation:**

We report a unique case of a Caucasian 65-year-old patient who was found to have vitreomacular traction in the left eye associated with progressive blurry vision. The patient’s vitreomacular traction unexpectedly resolved following a deep scuba dive to 60 m. The patient experienced an acute onset of floaters followed by a marked improvement in visual acuity. Spectral-domain optical coherence tomography confirmed complete resolution of vitreomacular traction without residual traction or macular damage. Although the effects of barometric pressure changes are well documented in postoperative vitrectomy patients with intraocular gas tamponade, little is known regarding their impact on untreated vitreomacular traction.

**Conclusions:**

This case demonstrates a temporal association between extreme pressure fluctuations during deep diving and spontaneous release of vitreomacular traction. Further investigation into the effects of environmental barometric pressure on vitreomacular traction is warranted.

## Background

Vitreomacular traction (VMT) syndrome arises due to incomplete posterior vitreous detachment (PVD), wherein persistent adhesion at the macula results in anteroposterior traction on the retinal surface. This mechanical stress can lead to visual distortion, cystoid macular edema (CME), and, in severe cases, progression to a full-thickness macular hole [1]. Although some cases resolve spontaneously, the reported rate of spontaneous resolution is approximately 11% [2]. When symptoms are persistent or progressive, pars plana vitrectomy or pharmacologic vitreolysis is the standard approach to relieve traction and restore macular architecture.

This case report presents an unusual instance of VMT resolution following deep-sea diving, raising the possibility that barometric pressure fluctuations may play a role in vitreoretinal dynamics.

## Case presentation

### Initial evaluation

**Fig. 1:**
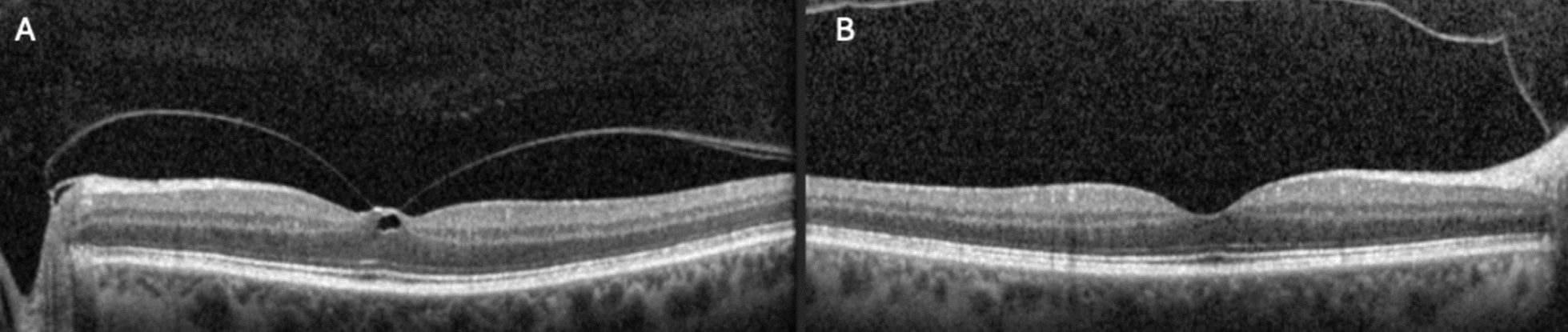
**A** SD-OCT revealing focal VMT in the left eye, without evidence of CME or macular hole formation. **B** SD-OCT in the right eye was unremarkable without evidence of VMT. *SD-OCT* spectral domain optical coherence tomography, *VMT* vitreomacular traction, *CME* cystoid macular edema

A 65-year-old Caucasian patient presented with progressive blurry vision in their left eye (OS) over several months. The patient’s past medical history was unremarkable aside from mild nuclear sclerosis in both eyes. Medical, family, psychosocial, and genetic history were non-contributory. Ocular history was unremarkable, with no previous ophthalmologic intervention. Visual acuity was recorded as 20/30 (pinhole to 20/25) in the right eye, and 20/40 (pinhole to 20/30) in the left eye. Spectral-domain optical coherence tomography (SD-OCT) revealed focal VMT in the left eye, without evidence of CME or macular hole formation (Fig. 1A). SD-OCT in the right eye was unremarkable (Fig. 1B). The patient’s bilateral nuclear sclerosis was stable from previous examinations. The patient was counseled on therapeutic options, including surgery to release the VMT or observation. Given their preserved visual acuity, observation was recommended, with follow-up scheduled in three months.

Five months later, the patient’s symptoms persisted, with no interval changes on SD-OCT or visual acuity. During this visit, the patient mentioned an upcoming scuba diving trip in which they planned to descend to depths of 60 m (~ 197 feet). Given the lack of literature on the effects of extreme underwater pressure changes on VMT, the patient was advised to proceed with caution.

**Fig. 2:**
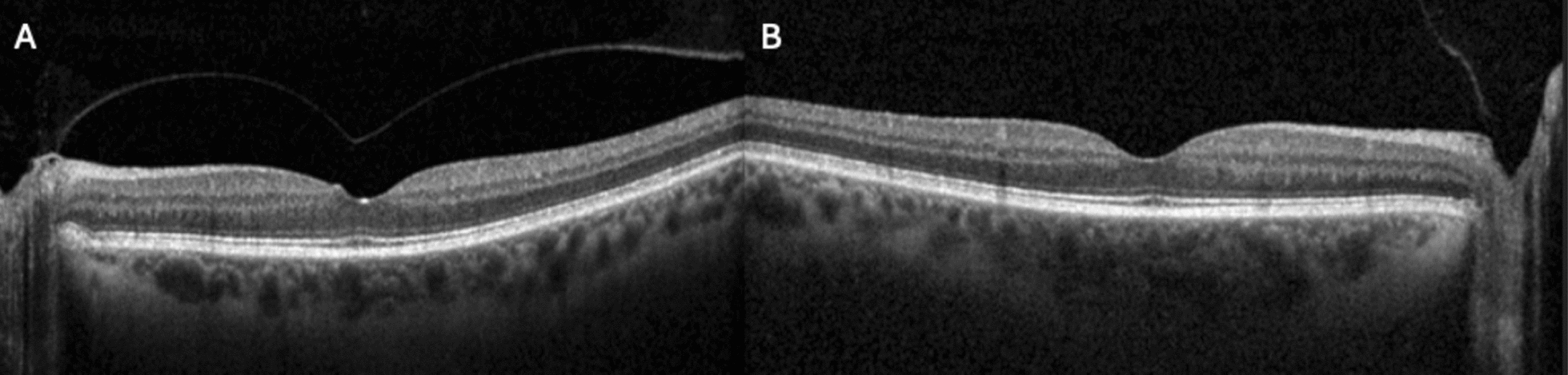
**A** Repeat SD-OCT demonstrated complete resolution of the VMT in OS, with no residual traction or evidence of CME (**A**). **B** SD-OCT of right eye was unchanged. *SD-OCT* spectral domain optical coherence tomography, *VMT* vitreomacular traction, *CME* cystoid macular edema

Upon returning from their trip, the patient reported a noticeable improvement in vision, preceded by the sudden onset of floaters. Although the patient’s visual acuity was slightly worse in right eye (20/50, pinhole to 20/20), their visual acuity in the left eye had improved to 20/20. Repeat SD-OCT demonstrated complete resolution of the VMT in the left eye, with no residual traction, macular hole, or evidence of CME (Fig. 2A). Repeat SD-OCT in the right eye was unchanged (Fig. 2B). The exam was otherwise non-contributory, with stable nuclear sclerosis in both eyes.

## Discussion

Spontaneous resolution of VMT is most commonly attributed to progressive vitreous liquefaction or completion of a PVD over time, and may occur without any identifiable external trigger [3]. Studies suggest that broad vitreoretinal adhesions are less likely to resolve spontaneously compared to focal adhesions [4]. While this patient had a focal VMT, clinical management typically involves observation for six months before considering surgical intervention if no resolution occurs. At her initial visit, she had already been experiencing symptoms for two months, and by the time of her five-month follow-up, it was clear that her symptoms had persisted beyond the six-month observation period. However, given her upcoming travel plans, the discussion regarding surgical intervention was deferred until her return. Notably, the timing of VMT resolution temporally coincided with exposure to extreme barometric pressure fluctuations during deep diving, although a causal relationship cannot be established from a single case.

The effects of pressure changes on intraocular structures are well-documented in vitrectomized patients with intraocular gas tamponade, where decreased atmospheric pressure during air travel or high-altitude ascent leads to intraocular gas expansion and significant intraocular pressure (IOP) elevation [5]. One study reported a significant increase in IOP per 1,000 feet of ascent in a patient with 50% perfluoropropane (C_3_F_8_) gas fill, reaching an IOP of 42 mmHg at 2,600 feet [6]. However, the impact of increased pressure at deep-sea levels on the vitreous body remains unclear.

At 60 m, ambient pressure is approximately seven times normal atmospheric pressure. Although a direct causal relationship cannot be established, several physiologically plausible mechanisms may explain how pressure changes during deep diving could influence vitreomacular adhesion. Importantly, the eye is not directly exposed to ambient water pressure because a properly equalized diving mask maintains near-atmospheric pressure over the ocular surface. Therefore, any effects are likely mediated indirectly through systemic or intraocular physiologic changes rather than external globe compression.

Rapid ascent from depth can produce nitrogen supersaturation and systemic decompression stress, which has well-described effects on tissues throughout the body [7]. Prior reports have documented ocular manifestations in this setting, including intraocular bubble formation and posterior vitreous detachment, suggesting that pressure-related physiologic stress may influence the vitreoretinal interface [8]. Experimental and environmental pressure studies further support that alterations in ambient pressure can affect intraocular structures and ocular biomechanics [9]. Although these processes typically present with systemic symptoms rather than isolated ocular findings, they provide biologic plausibility that rapid pressure transitions could contribute to vitreoretinal separation in susceptible individuals.

In addition, exposure to hyperbaric environments has been associated with modest reductions in intraocular pressure, although the mechanisms remain incompletely understood and may relate to altered episcleral venous pressure, choroidal blood volume, or aqueous humor dynamics [10]. Transient intraocular pressure fluctuations during descent and ascent could theoretically modify tractional forces at focal vitreomacular adhesions or facilitate redistribution of liquefied vitreous, thereby promoting separation. In the present case, the absence of systemic features of decompression illness makes localized intraocular gas formation unlikely; however, pressure-related physiologic changes may still have contributed indirectly to vitreoretinal detachment [11].

Taken together, these considerations suggest that transient pressure-related alterations in intraocular physiology and vitreous biomechanics may have facilitated completion of a posterior vitreous detachment in this eye. These mechanisms remain speculative and are presented to provide physiologic context rather than imply causation, as spontaneous resolution independent of diving remains equally plausible.

While causality cannot be established, the temporal association between deep diving and VMT resolution should be interpreted cautiously. As a single case report, this observation is hypothesis-generating rather than confirmatory and cannot determine whether pressure exposure directly influenced vitreoretinal separation. The proposed mechanisms are supported primarily by physiologic plausibility and related literature on environmental and decompression effects on ocular structures, rather than direct experimental evidence. Spontaneous resolution independent of diving remains equally plausible. Further investigation in larger cohorts or controlled settings would be necessary to clarify whether hyperbaric pressure exposure meaningfully affects vitreoretinal dynamics.

## Conclusion

This case highlights a temporal association between extreme environmental pressure exposure and spontaneous VMT resolution. Although the mechanism remains uncertain and causality cannot be inferred from a single observation, this report raises the possibility that barometric fluctuations may influence vitreoretinal dynamics. Further study is needed to better characterize the effects of environmental pressure changes on the vitreoretinal interface.

## Data Availability

Not applicable.

## Abbreviations

CME: Cystoid macular edema
IOP: Intraocular pressure
OS: Left eye (Oculus sinister)
OD: Right eye (Oculus dexter)
PVD: Posterior vitreous detachment
SD-OCT: Spectral-domain optical coherence tomography
VMT: Vitreomacular traction
